# Evaluating disinfectant efficacy on mixed biofilms comprising Shiga toxigenic *Escherichia coli*, lactic acid bacteria, and spoilage microorganisms

**DOI:** 10.3389/fmicb.2024.1360645

**Published:** 2024-04-03

**Authors:** Kavitha Koti, Argenis Rodas-Gonzalez, Celine Nadon, Tim McAllister, Xianqin Yang, Claudia Narváez-Bravo

**Affiliations:** ^1^Department of Food and Human Nutritional Science, University of Manitoba, Winnipeg, MB, Canada; ^2^Department of Animal Science, University of Manitoba, Winnipeg, MB, Canada; ^3^National Microbiology Laboratory, Public Health Agency of Canada, Winnipeg, MB, Canada; ^4^Agriculture and Agri-Food Canada, Lethbridge Research and Development Centre, Lethbridge, AB, Canada; ^5^Agriculture and Agri-Food Canada, Lacombe Research and Development Centre, Lacombe, AB, Canada

**Keywords:** meat processing facilities, beef, lactic acid bacteria, spoilage bacteria, whole genome sequencing, Shiga toxin-producing *Escherichia coli*, biofilm

## Abstract

This study aimed to investigate the impact of temperature and the presence of other microorganisms on the susceptibility of STEC to biocides. Mature biofilms were formed at both 10°C and 25°C. An inoculum of planktonic bacteria comprising 10^6^ CFU/mL of spoilage bacteria and 10^3^ CFU/mL of a single *E. coli* strain (O157, O111, O103, and O12) was used to form mixed biofilms. The following bacterial combinations were tested: T1: *Carnobacterium piscicola* + *Lactobacillus bulgaricus* + STEC, T2: *Comamonas koreensis* + *Raoultella terrigena* + STEC, and T3: *Pseudomonas aeruginosa* + *C. koreensis* + STEC. Tested biocides included quaternary ammonium compounds (Quats), sodium hypochlorite (Shypo), sodium hydroxide (SHyd), hydrogen peroxide (HyP), and BioDestroy®-organic peroxyacetic acid (PAA). Biocides were applied to 6-day-old biofilms. Minimum Bactericidal Concentrations (MBC) and Biofilm Eradication Concentrations (BEC) were determined. Planktonic cells and single-species biofilms exhibited greater susceptibility to sanitizers (*p* < 0.0001). *Lactobacillus* and *Carnobacterium* were more susceptible than the rest of the tested bacteria (p < 0.0001). Single species biofilms formed by *E. coli* O111, O121, O157, and O45 showed resistance (100%) to Shypo sanitizer (200 ppm) at 25°C. From the most effective to the least effective, sanitizer performance on single-species biofilms was PAA > Quats > HyP > SHyd > Shypo. In multi-species biofilms, spoilage bacteria within T1, T2, and T3 biofilms showed elevated resistance to SHyd (30%), followed by quats (23.25%), HyP (15.41%), SHypo (9.70%), and BioDestroy® (3.42%; *p* < 0.0001). Within T1, T2, and T3, the combined STEC strains exhibited superior survival to Quats (23.91%), followed by HyP (19.57%), SHypo (18.12%), SHyd (16.67%), and BioDestroy® (4.35%; *p* < 0.0001). O157:H7-R508 strains were less tolerant to Quats and Shypo when combined with T2 and T3 (*p* < 0.0001). O157:H7 and O103:H2 strains in mixed biofilms T1, T2, and T3 exhibited higher biocide resistance than the weak biofilm former, O145:H2 (*p* < 0.0001). The study shows that STEC within multi-species biofilms’ are more tolerant to disinfectants.

## Introduction

Despite numerous efforts to implement food safety measures to reduce Shiga toxin-producing *E. coli* (STEC) related outbreaks, their persistence continues to impact the safety of the food supply in North America. While the O157 serogroup is frequently linked to diseases, non-O157 STEC O26, O45, O103, O111, O121, and O145 have emerged as notable contributors to global foodborne illnesses (Wang et al., 2012). In Canada, the incidence of non-O157 increased significantly in 2019 to 2.5 cases per 100,000, while O157:H7 has remained constant at 1.06 cases per 100,000 since 2010 (Canada, 2016; Ma et al., 2019).

The most common illness associated with STEC is gastroenteritis, but in some cases, more severe and potentially life-threatening conditions such as hemolytic uremic syndrome can develop (Smith et al., 2014). One family of STEC virulence factors is the Shiga toxins (Stx). The Stx family consists of a group of heterogeneous toxins, Stx1 and Stx2. STEC strains that carry the gene for Stx2 cause more severe diseases, such as hemolytic uremic syndrome, than strains that carry Stx1 or both Stx1 and Stx2 (Melton-Celsa et al., 2007). Cattle and other ruminants can be carriers of STEC, and the consumption of contaminated beef products has been often identified as the source of disease outbreaks (Beier et al., 2016; CDC, 2023). Within the food industry, STEC can attach to surfaces and form biofilms (Wang et al., 2012), which is likely impacting their ability to survive in a variety of environments, including those associated with food processing.

The formation of biofilms presents a significant challenge in the food industry. Biofilms can serve as potential reservoirs of foodborne pathogens and spoilage microorganisms. Furthermore, bacteria within biofilms can detach and contaminate other surfaces, including food, resulting in cross-contamination (Marouani-Gadri et al., 2009; Silagyi et al., 2009; Wang et al., 2012). Within meat processing plants, biofilms can be formed by a diversity of bacteria, including spoilage bacteria and pathogens (Vogeleer et al., 2014; Yang et al., 2018; Visvalingam et al., 2019). Some of the spoilage microorganisms reported to be dominant in meats and meat processing establishments include *Pseudomonas*, *Carnobacterium, Brochothrix, Serratia, Kocuria, Leuconostoc, Aerococcus, Pediococcus, Yersinia, Corynebacterium, Escherichia, Micrococcus*, and *Lactobacillus* (Casaburi et al., 2011; Wang et al., 2014, 2016). Lactic acid bacteria are considered spoilage bacteria when present in fresh beef products, but can produce various antimicrobial compounds, such as bacteriocins, hydrogen peroxide, and organic acids which can inhibit the growth of foodborne pathogens (Perez et al., 2014).

The elimination of biofilms formed by bacteria, including STEC and spoilage microorganisms, remains a challenge for the food industry (Chapman, 2003; Fouladkhah et al., 2013). While there has been extensive research on the impact of STEC biofilms on food safety, there is limited information available on the impact of spoilage microorganisms that form biofilms alongside STEC. Additionally, there is a lack of knowledge on the environmental factors such as temperature that affect biofilm formation, and the ability of STEC to survive biocides. The objective of this research was to evaluate a number of commonly used disinfectants in the food industry for their minimum bactericidal concentration (MBC) and biofilm eradication concentration (BEC) of multispecies STEC biofilms at 10°C and 25°C. Additionally, we investigate the genetic composition of STEC in terms of antimicrobial resistance and biofilm formation genes.

## Materials and methods

### Bacterial strain selection and culture conditions

A total of nine STEC, four spoilage bacteria (SP) and six lactic acid bacteria (LAB) strains were included in this study (Table 1). The bacterial cultures were maintained in Trypticase Soy Broth (TSB; Becton, Dickinson and Company, MD, United States) supplemented with 15% glycerol and stored at −80°C. Before the experiments, each stock culture was plated on trypticase soy agar (TSA; Difco Becton, Dickinson and Company, MD, United States) since all tested strains can grow well in this media, STEC strains were cultured on MacConkey agar plates (Hardy Diagnostics Inc., Santa Maria, CA, United States). A single colony of each bacterial strain was transferred from each plate into 5 mL of TSB and incubated at 37°C for 18 to 24 h. Cells were harvested by centrifugation (4500xG) for 5 min at room temperature. After centrifugation, the supernatant was decanted, and the pellet was resuspended in 5 mL sterile Butterfield’s Phosphate Buffer (BPB; Hardy Diagnostics Inc., Santa Maria, CA, United States). This procedure was repeated three times for three wash steps in BPB. Each bacterial suspension was adjusted to a final concentration of 10^8^ colony-forming units (CFU/mL) using a 0.5 McFarland Standard and further diluted in Lennox Broth no-salt LB-NS broth (LB-NS; Tryptone 10 g/L and yeast extract 5 g/L) to achieve a concentration of 10^6^ CFU/mL.

**Table 1:** STEC, LAB, and spoilage bacteria used in this study.

Serotype	Strain ID	Source	Category
O26: H11	00-3941	Human	STEC
O45: H7	05-6545	Human	STEC
O103: H2	99-2076	Human	STEC
O111: NM	CFS3	Human	STEC
O121: H19	03-2832	Human	STEC
O145: H2	75-83	Human	STEC
O157: H7	1934	Beef	STEC
O157: H7	1931	Hamburger	STEC
O157: H7	R508	Bovine/feces	STEC
*Lactobacillus sakei*	S19	Vacuum-packaged meat	LAB
*Carnobacterium divergens*	B1	Vacuum-packaged meat	LAB
*Lactobacillus delbrueckii* subsp. *bulgaricus*	ATCC11842	Yogurt	LAB
*Lactobacillus curvatus*	133 L	Meat Starter Culture	LAB
*Lactobacillus sakei*	LB 808 (S206)	Unknown	LAB
*Carnobacterium* sp.	M5L1	Vacuum package pork	LAB
*Carnobacterium divergens*	ATCC 35677	Vacuum package of minced beef	LAB
*Serratia* sp.	S10	Vacuum-packaged meat	Spoilage
*Comamonas* sp.	25_64	Meatpacking plant	Spoilage
*Raoultella* sp.	ENT25_16	Meatpacking plant	Spoilage
*Pseudomonas aeruginosa*	ATCC 7700	Well water	Spoilage

### Biocide solutions

Six disinfectants commonly employed in the food industry were evaluated. Disinfectants were prepared according to the manufacturer’s specified concentrations (Table 2). Stock solutions were prepared in sterile hot water (50°C) and maintained at 40°C to 50°C. Solutions were used within 30 min after preparation. Before initiating testing, the concentration of active chlorine in the sodium hypochlorite solution was tested using a chlorine analysis method (Total) Test Kit (HACH, Model CN-65, Ontario, Canada).

**Table 2:** Biocides and manufacture recommended concentrations.

Trade name	Active ingredients	Acronyms	Stock	Dilution	Use level	Contact time	Temperature
Chloreco	Sodium hypochlorite	Shypo	12%	1:100	Sanitizing: 200 ppm (no rinse needed)	< 5 vary min	5°C–65°C
					Surface sanitizing: 1,200 to 120,000 ppm (must be rinsed with water)	5–30 min	
Caustek 50	Sodium hydroxide	Shyd	50%	1:200–1:9	2,500 ppm (must be rinsed with water)	15–40 min.	50°C–100°C (Optimal:75°C–85°C)
Powerquat	Quaternary ammonium, C12-18-alkyl[(ethyl phenyl)methyl] dimethyl, chlorides (5%)	Quats (PQ)	10%	22:4,000	200 ppm (no rinse) 550 ppm	10 min	Not specified
	Quaternary ammonium, benzyl-C12-18-alkyl dimethyl, chlorides and ethanol (5%)						
Oxygerm	Hydrogen peroxide 21.7% Peracetic acid 5.1.%	HyP	5%	1:200	250 ppm (must be rinsed)	Vary	5°C–40°C
Germarc	Quaternary ammonium compounds, benzyl-C12-16-alkyl dimethyl, chlorides and ethanol	Quats (GM)	10%	1:160	Sanitation: 200 ppm	Up to 10 min	5°C–65°C
					Disinfection: 400–600 ppm		
Biodestroy	Hydrogen peroxide, dodecylbenzene sulphonic acid, Acetic acid, Peroxyacetic acid Alcohols, C12-15, ethoxylated	PAA	6%	1:100	600 ppm	5 min	Not specified

^*^All sanitizers were obtained from SANI MARC®. Two types of Quats are used for this experiment, Powerquat and Germarc. Powerquat is specially designed for food processing plants where disinfection is of prime importance and mostly used on hard surfaces, and proved to be an effective bactericide in the presence of organic soil. In contrast, Germarc is commonly used as a sanitizer on all surfaces with N-Alkyl (50% C14, 40% C12, 10% C16) dimethyl benzyl ammonium chloride: 10.0% and its effectiveness is affected by the presence of organic soils and needs higher exposure time.

### Minimum bactericidal concentration

A total of nine STEC strains, four spoilage and six lactic acid bacteria were included to determine their minimal bactericidal concentration. Minimum bactericidal concentration (MBC) was determined using 96-well microplates to establish the lowest concentration of biocide required to kill 99.9% of the bacterial population over a fixed contact time (Rodríguez-Melcón et al., 2021). Before conducting the experiments, various concentrations of each biocide were assessed to establish the specific concentration range that should be focused on as a starting point. To determine MBC values of all the biocides for each strain were determined as described by Lambert and Pearson (2000) with a few modifications. Briefly, biocide dilution series were prepared in 96-microplates (Nunc, flat-bottom non-treated, Roskilde, Denmark) with each well filled with 180 μL of diluted antimicrobial agent followed by 20 μL of a culture suspension containing 10^6^ CFU/mL. Each plate included one column as a negative control that contained only Buffered Peptone Water (BPW, Hardy Diagnostics Inc.) and a positive control that contained only bacteria without biocide. The biocides were allowed to sit for 10 min. After 10 min, Dey/Engley broth (22 μL; BBL, Difco, Sparks, MD) was added into the wells to neutralize the biocide activity and allowed to stand for at least 5 min. Subsequently, the spot plate technique was used, with five—5 μL of the solution was plated on agar. STEC strains were cultured on MacConkey agar overlayed with TSA while SP and LAB were cultured on TSA, Lactobacilli MRS agar (Difco) and Pseudomonas agar. All plates were incubated at 25°C for 72 h. MBC was defined as the lowest concentration of a biocide at which no bacterial growth is observed. There were two replicate microplates per trial, with independent experiments being conducted three times.

### Selection of strains for multispecies biofilms

STEC strains [*E. coli* O145 (75–83), *E. coli* O157:H7 (1934), *E. coli* O103:H2 (99-2076) and *E. coli* O157:H7 (R508)] were included in the multispecies biofilms section. These strains were selected based on their biofilm-forming abilities, expression of genes involved in curli and cellulose synthesis at 25°C, as well as their MBC. *E. coli* O145 (75–83) is a weak biofilm former with no expression of genes associated with curli or cellulose synthesis. *E. coli* O157:H7 (1934): is an intermediate biofilm former but did not synthetized curli or cellulose. *E. coli* O103:H2 (99-2076) and *E. coli* O157:H7 (R508) are strong biofilm formers and display both curli and cellulose phenotypes. The LAB strains, *Carnobacterium piscicola* and *Lactobacillus delbrueckii* subsp. bulgaricus ATCC 11842, could be considered spoilage bacteria, but we also had an interest in the impact that these LAB have on the susceptibility of STEC within biofilms to biocides. *Pseudomonas aeruginosa* was included as a common biofilm forming spoilage bacteria found in food processing environments (Thi et al., 2020) and its tolerance to disinfectants (Sagripanti and Bonifacino, 2000). In addition, there are concerns that *Pseudomona*s may disseminate antimicrobial resistance genes through horizontal gene transfer (Quintieri et al., 2019).

The selection process also considered MBC values; O145:H2 (75–83) exhibited the highest MBC for all biocides, followed by O157:H7 (R508). Strain 1934 (O157:H7) displayed higher MBC to hydrogen peroxide than other strains, while R508 exhibited high MBC values to Quaternary Ammonium Compounds (Quats). In previous research by our group (Nan et al., 2022) we found that T1, T2, and T3 biofilm combinations are capable of forming multispecies biofilms with O103:H2 (99-2076) and O157:H7 (1934), which promote survival and their transfer to beef.

Bacterial strains were combined as follows: LAB T1: *Carnobacterium piscicola* + *Lactobacillus bulgaricus +* individual STEC strain and spoilage bacteria combination T2: *Comamonas koreensis* + *Raoultella terrigena* + individual STEC strain and spoilage bacteria combination T3: *Pseudomonas aeruginosa* + *C. koreensis* + individual STEC strain. Four selected STECs (O157-R508, O157-1934, O145-75-83 and O103-992075) were added individually to mixed pre-formed biofilms T1, T2, and T3.

### Single-species and multispecies biofilm formation

For single-species biofilms, harvested cells of each strain were diluted in LB-NS broth supplemented with 16.7% filter sterilized beef purge (10% v/v; Nan et al., 2022) and 180 μL dilute was added to each well in a 96-well microplate to achieve a final population of 10^6^ CFU/mL (Poimenidou et al., 2016). The microplates were incubated at 10 and 25°C for six d. For multispecies biofilms, spoilage bacteria or LAB were cultured in 100 mL sterile glass bottles containing LB-NS broth supplemented with sterilized beef purge. Fresh cultures of each specific spoilage and lactic acid bacteria (LAB) were introduced into LB-NS broth and allowed to reach 10^6^ CFU/mL. Cultures were mixed, and 180 μL of the mixture were transferred into each well in a 96-well microplate. Negative controls consisting only of LB-NS broth supplemented with beef purge were included in each plate. Controls that consisted of each of the individual bacterial strains were also included. Microplates were incubated at 10°C and 25°C for six days. After this period, using a multichannel pipette, the microplates were washed three times with 300 μL BPB to remove the loosely attached cells, with the supernatant being aspirated after each washing. Subsequently, fresh STEC culture was diluted in LB-NS broth with beef purge to achieve 10^3^ CFU/mL and introduced into wells with pre-formed biofilms. Positive control wells (T4), contained only STEC strains, while negative control wells contained only culture media. Microplates were incubated at 10 and 25°C for an additional 6 days after which each well was washed three times with 300 μL of Butterfield’s Phosphate Buffer (BPB). After washing, microplates were air-dried for 30 min. and then stored for an additional 6 d at their original respective temperature (i.e., 10°C or 25°C). During the experiment, three groups of microplates were included in each experiment. The first was employed in the crystal violet assay, the second was used to enumerate bacteria and the third was used to assess biocide eradication. Spoilage and *E. coli* were enumerated through serial dilutions (1:10) on selective agars, MacConkey (Criterion, Hardy Diagnostics, Santa Maria, CA, United States), MRS (Oxoid Ltd., Thermo Fisher. Hampshire, United Kingdom) and Pseudomonas agar + selective supplement (Oxoid-ThermoFisher, Nepean, ON). Positive controls of single-species *E. coli* and spoilage bacteria were also enumerated to compare their growth to that observed in multi-species biofilms. Experiments were carried out in two independent biological experiments with three technical replicates each.

### Biofilm assessment using the crystal violet method

To assess biofilm development, 200 μL of methanol was pipetted into wells containing pre-formed biofilms and allowed to stand for 15 min. Methanol was then aspirated using a microplate washer (405 LS, BioTek, Winooski, VT, United States), and 200 μL of 0.1% crystal violet (CV; Sigma Aldrich) was added to each well and allowed to stand for an additional 15 min. Microplates were then washed three times with 300 μL BPB per well. Residual crystal violet was solubilized in 200 μL of 85% ethanol. Biofilm forming ability was determined indirectly by measuring the level of residual chromophore using a microplate reader at 630 nm (BioTek ELx800; BioTek Instruments Inc., Winooski, VT, United States). The experiment was repeated three times in duplicate for each strain combination. Biofilm-forming ability was estimated using optical density cutoffs (ODc) as described by Adator et al. (2018).

### Biofilm eradication concentration of single-species and multispecies biofilms

Biocide concentrations tested in this study were as per recommended by the manufacturer and include sodium hypochlorite (Shypo) at 1,200 ppm, sodium hydroxide (Shyd) at 2,500 ppm, Quat’s Power Quat (PQ) at 550 ppm, Quats Germarc (GM) at 600 ppm, hydrogen peroxide at 250 ppm and peroxyacetic acid alcohols Biodestroy (PAA) at 600 ppm (Table 2). Sanitizer dilutions were prepared in 50°C sterile distilled H_2_O (dH_2_O) water. Designated wells containing either single or multi-species biofilms within a 96-well microplate were filled with 200 μL of each biocide at its respective concentration. Contact was allowed for 10 min, with biocides subsequently neutralized by adding 22 μL of Dey/Engley broth and allowing it to stand for 5 min. The supernatant was aspirated using a multichannel pipette and washed thrice with 200 μL BPB. Afterwards, 200 μL of buffered peptone water (BPW) was added to all wells and the surface of each well was scraped using a sterile wooden toothpick. Then the toothpick was removed making sure that no biofilms was attached to it, then the microplate was sonicated at 40 kHz for 1 min (Branson 2800, Branson Ultrasonics Co., Danbury, CT, United States). Bacterial survival was assessed using the spot plate technique using McConkey, MRS and Pseudomonas agars overlayed with 10 mL of TSA. McConkey agar was used to assess STEC viability, while SP and LAB bacteria were cultured on TSA, Pseudomonas and MRS agar, respectively. Each type of agar was inoculated with five 10 μL drops of BPW obtained from each microplate well in duplicate, followed by incubation at 25°C for 72 h. The biofilm eradication concentration was calculated as the lowest biocide concentration that prevented bacterial growth. The experiments were conducted three times.

### Whole genome sequencing and bioinformatics analysis

Genomic DNA from STECs strains (O157:H7 R508, 1,034, O103 and O145) was extracted using the DNeasy Blood and tissue kit (Qiagen, Inc., Toronto, Ontario, Canada) following manufacturer recommendations for Gram negative bacteria. DNA concentration and quality was tested using NanoDrop (NanoDrop technologies, Wilmington, United States).

Whole genomic data of the isolates was acquired by 150 bp paired-end sequencing on the Illumina MiSeq using MiSeq Reagent Kit V2 300 cycles. The library samples were prepared using Nextera XT DNA sample preparation kit. Data was assembled into contigs using SPAdes assembler (v3.0). Contigs less than 1 kb and coverage less than ×15 were filtered out and the remainder were annotated with Prokka (v1.3).

Comparative genomics was employed to study the genetic differences among STEC strains (O157:H7, O103:H2, and O145:H2; Supplementary Table S1) concerning their biofilm formation and biocide resistance. Using RASTtk4 annotation, we focused on intra-genus comparisons (PLfams; Overbeek et al., 2005, 2014). *Escherichia coli* K12 substr. MG1655 served as the reference genome. Genomes were included based on quality: completeness >96% and minimal contamination <2% (Supplementary Table S1). Bioinformatic analyses were performed using pipelines from the BV-BRC website.^1^

### Statistical analysis

Biofilm-forming ability, optical density cutoffs (ODc) were calculated as three standard deviations from the mean value of the control negative, as described by Adator et al. (2018). Classifications included OD ≤ ODc = non-biofilm former; ODc < OD ≤ 2ODc = weak biofilm former; 2ODc < OD ≤ 4ODc = intermediate biofilm former; 4ODc < OD = strong biofilm former.

A frequency analysis was performed to determine the STEC, spoilage and LAB susceptibility or resistance in planktonic and single-species biofilm at 10°C and 25°C to the different biocides. Chi-square (χ2) analysis (Fisher’s exact test) was used for testing differences in frequencies of *E. coli* survival in multispecies biofilm among temperatures within a biocide.

All experiments were performed three times. The Proc Mixed procedure of the Statistical Analysis System (Cary, NC, United States) was used to analyze the data with the least mean separation accomplished using the PDIFF option. To compare optical density, and individual *E. coli* strains and spoilage bacteria count, a factorial model was applied to analyze the main effects of multispecies biofilms treatments, temperature, and their interaction.

## Results and discussion

### Minimum bactericidal concentration

In their planktonic state, all strains were susceptible to all biocides, except for some STEC strains that were insensitive to sodium hypochlorite when used in a concentration of 200 ppm. In general, lactic acid bacteria were more sensitive to disinfectants than STEC and spoilage bacteria.

Among the sanitizers, Biodestroy® (an organic peroxy acid) was the most effective, followed by quaternary ammonium compounds (quats), hydrogen peroxide, sodium hydroxide, and sodium hypochlorite (Table 3). Notably, the ppm required to prevent the growth of planktonic bacteria was considerably lower than the manufacturer’s recommended concentrations (Table 2). For instance, the MBC (minimum bactericidal concentration) of Biodestroy for STEC ranged from 20 to 26 ppm, whereas the manufacturer’s recommendation was 600 ppm. At the same time, LAB and SP required even lower concentrations, ranging from 13.3 to 16.7 ppm, with the exception of *Comamonas* (21.7 ppm) and *Pseudomonas* (23.3 ppm).

**Table 3:** Mean (PPM) of minimum bactericidal concentration of planktonic bacteria after treatment with different biocides at 25°C.

Bacteria	Biodestroy (organic peroxy acid)	Germarc (quats)	Oxygerm (hydrogen peroxide)	PowerQuat (quats)	Caustek (sodium hydroxide)	Chloreco (sodium hypochlorite)
*O145*: *H2*	26.0	159.0	31.7	100.0	630.0	216.7
*O121: H19*	21.0	153.3	44.5	96.7	616.7	335.0
*O157:H7 1934*	23.7	156.7	45.0	73.3	563.3	276.7
*O157:H7 1931*	24.3	148.3	36.7	83.3	623.3	435.0
*O26: H11*	22.3	146.7	28.3	86.7	636.7	208.3
*O157:H7 R508*	22.3	151.7	30.0	100.0	616.7	316.7
*O45: H7*	22.0	153.3	40.0	100.0	643.3	208.3
*O111: NM*	27.7	150.0	28.3	86.7	603.3	173.3
*O103: H2*	20.0	153.3	31.7	76.7	596.7	436.7
*L. sakei S19*	16.7	88.3	16.7	103.3	566.7	193.3
*Carnobacterium divergens*	13.3	95.0	21.7	90.0	613.3	96.7
*Lactobacillus bulgaricus*	13.3	96.7	20.7	106.7	583.3	70.0
*Lactobacillus curvatus*	13.3	103.3	18.3	93.3	666.7	86.7
*Lactobacillus sakei*	16.7	91.7	16.7	93.3	723.3	93.3
*Carnobacterium piscicola*	16.7	95.0	23.3	93.3	490.0	63.3
*Carnobacterium divergens*	13.3	103.3	21.7	86.7	630.0	93.3
*Serratia* sp.	15.0	106.7	20.7	103.3	636.7	86.7
*Comamonas* sp.	21.7	95.0	22.3	90.0	690.0	116.7
*Raoultella* sp.	16.0	106.7	22.3	110.0	643.3	96.7
*Pseudomonas aeruginosa*	23.3	113.3	25.0	107.7	503.3	266.7

The proportion of resistant sessile strain to each sanitizer was calculated using a threshold value given by the manufacturer [organic peroxy acids = 600 ppm; sodium hydroxide = 2,500 ppm; sodium hypochlorite = 1,200 ppm; quats (GM) = 600 ppm; hydrogen peroxide = 250 ppm; Quats (PQ) = 550 ppm]. The mean difference is significant at the 0.05 level.

Regarding quaternary ammonium compounds, two distinct products, Germarc (GM) and Power Quat (PQ), were tested. Power Quat, a third-generation Quat, exhibited superior efficacy, requiring lower concentrations (73–103 ppm) for complete bacterial elimination compared to Germarc (88–159 ppm). As for hydrogen peroxide (Oxygerm), growth of STEC was inhibited at 28.3 to 45 ppm, while growth of spoilage and LAB was inhibited at 16.7 to 25 ppm. Sodium hydroxide at 490–666 ppm prevented the growth of all targeted bacteria.

In contrast, sodium hypochlorite (Chloreco) was less effective as concentrations higher than the manufacturer’s standard recommended concentration of 200 ppm were required to eradicate STEC strains (173.3–436.7 ppm) and *Pseudomonas aeruginosa* (266.7 ppm). The manufacture’s highest recommended concentration of sodium hypochlorite (1,200 ppm) was effective at killing all bacterial strains.

Many biocides have an optimum pH range of activity. For example, cationic biocides such as QACs (Quats) are most potent at alkaline pH, whereas hypochlorites are more effective at an acidic pH (Araújo, 2014). In our experiment, having the culture in BPW could have increased alkalinity and thus reduced the bactericidal activity of chlorine. In addition, bacterial growth in the 96-well microplates would result in the formation of organic matter such as beef purge that could have further inactivated free chlorine (Bloomfield and Uso, 1985).

Disinfectants often contain more than one type of chemical active ingredient, making it more difficult for microorganisms to develop resistance due to their different mechanisms of bacterial inhibition (McDonnell and Russell, 1999). Sodium hypochlorite is a potent oxidizing agent with broad-spectrum antimicrobial activity. Previous research targeting planktonic *E. coli* O157:H7 planktonic cells (10^8^ CFU/mL) exposed to 200 ppm of sodium hypochlorite declined to 5.1 logs after 10 min (Bridges et al., 2022). These findings are similar to ours, where viable STEC were still isolated after exposure to 200 ppm sodium hypochlorite for 10 min.

Regarding quaternary ammonium compounds, others report lower MBC concentrations for *E. coli*, ranging from 6.3 to 12.5 ppm (Castro et al., 2023). Previous research has documented instances where bacterial susceptibility to lower sanitizer concentrations than those recommended by the manufacturer has been observed, although different chemical sanitizers were tested (Sidhu et al., 2001).

There is a shortage of data regarding LAB and other spoilage bacteria susceptibility to biocidal agents used in food processing facilities. Much of the existing data on biocide resistance focuses on foodborne pathogens. Spoilage bacteria can form robust biofilms that reduce product shelf-life, and these communities may also harbor foodborne pathogens that threaten food safety. Consequently, it is also important to investigate the susceptibility of spoilage bacteria to biocides, crucial for the development of risk mitigation strategies aimed at preserving the quality and safety of food products.

### Crystal violet assay to identify the strongest multispecies biofilms

**Figure 1:**
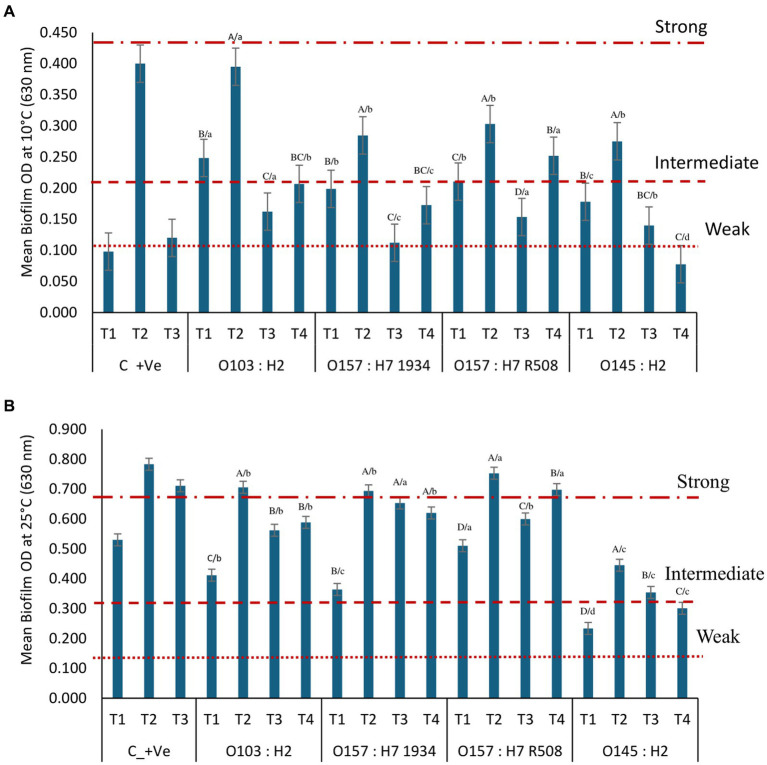
Optical density (OD) from STEC multispecies biofilm formation on microplates at 0 days at **(A)** 10^°^C and **(B)** 25°C according to their biofilm-forming ability with T1: *Carnobacterium piscicola* + *Lactobacillus bulgaricus*, T2: *Comamonas koreensis* + *Raoultella terrigena*; T3: *Pseudomonas aeruginosa* + *C. koreensis*, C_+ve are control positive values of T 1, T2, and T3 alone without STEC; T4: single species STEC. Biofilm-forming ability at 10°C: non-biofilm formers (OD ≤ 0.105); weak (OD > 0.105 to ≤0.210); intermediate (OD > 0.210 to ≤0.420); strong (OD > 0.420) biofilm formers. Biofilm-forming ability: at 25^°^C non-biofilm formers (OD ≤ 0.171); weak (OD > 0.171 to ≤0.342); intermediate (OD > 0.342 to 0.684); strong (OD > 0.684) biofilm formers. ABCD/, indicates significant differences (P ≤ 0.05) between multiple species biofilms treatment within each STEC strain. abcd, indicates significant differences (*P* ≤ 0.05) between STEC strains within the same multiple biofilm treatment.

**Figure 2:**
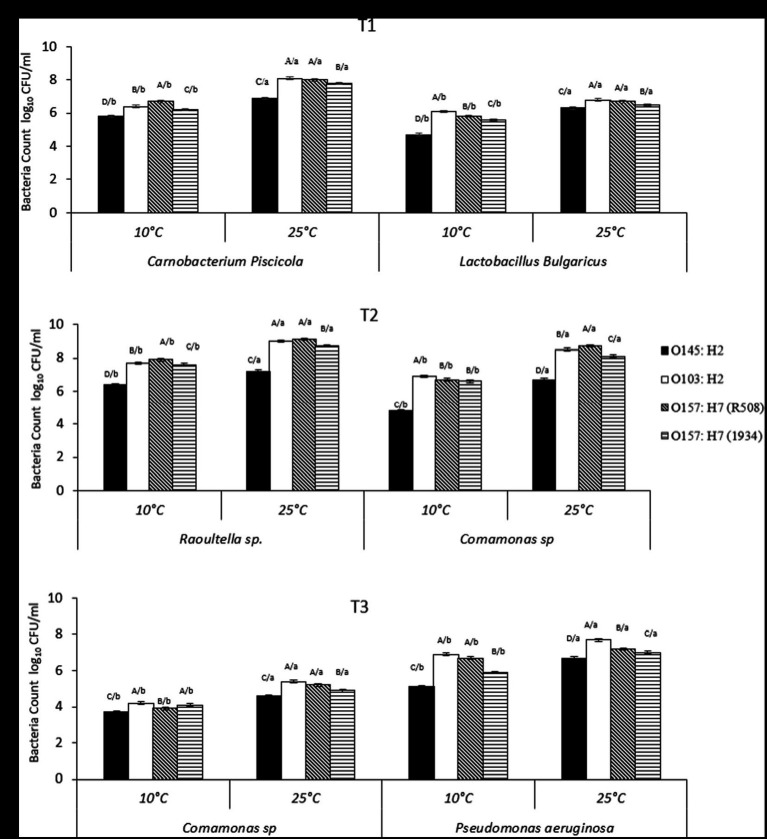
**(A–C)** Number of individual spoilage bacteria counts (CFU/mL) in multispecies biofilms at 10°C and 25°C, from Tl, T2, and T3, where Tl: *Camobactenum piscicola* + *Lactobacillus bulgaricus* + *Escherichia coli*; T2: *Raoultella* sp. + *Comamonas* sp. + *E. coli*; T3: *Pseudomonas aeruginosa* + *Comamonas* sp. + *E. coli*. The counts of spoilage bacteria were recorded when present with different types of *E. coli* (1934, O103, O145, R508). Effects of interaction of treatment (Tl, T2, T3) × *E. coli* (1934, O103, O145, R508) x temperature (10°C vs. 25°C; *p* < 0.01). ABCD/, indicates significant differences (*P* ≤ 0.05) STEC strains within the same temperature and spoilage bacteria. /abcd, indicates significant differences (*P* ≤ 0.05) between different temperatures within STEC strain and spoilage bacteria.

At 25°C, biofilm combinations T2 (*Comamonas koreensis*, *Raoultella terrigena* + STEC) and T3 (*Pseudomonas aeruginosa*, *C. koreensis* + STEC) produced stronger biofilms compared to those produced by *Lactobacillus + Carnobacterium* + STEC (T1; Figures 1A,B). Among the STEC single species strains (T4), O157 (R508 and 1934) and O103 formed more robust biofilms than O145:H2. Addition of STEC to biofilms, particularly O145:H2, appeared to weaken biofilm formation (*p* < 0.001). *E. coli* O145 also negatively impacted LAB and spoilage bacterial populations within biofilms at 25°C (*p* < 0.001; Figure 2). In contrast, O157:H7 (R508), appeared to enhance biofilm formation when combined with T2, whereas O157-1934 enhanced biofilm formation when combined with T2 and T3 (Figures 1A,B). Our previous observations in this study on single-species biofilms (data not shown) showed that O157 (R508) formed the most robust biofilms, followed by 1934 and O103, which formed intermediate biofilms. These findings suggest that STEC may contribute EPS to the formation of multispecies biofilms.

At 10°C, T1 + STEC and T3 + STEC and controls (T4) formed weaker biofilms. In contrast, combination T2, consisting of *Comamonas, Raoultella*, and STEC, formed intermediate biofilms alone or in combination with O103:H2, O157:H7 (R508) and O145. *Raoultella terrigena* is a member of the *Enterobacteriacea* family and exhibits similarities to *Klebsiella*. It is typically categorized as a mesophilic bacterium with optimal growth at 35°C to 37°C. However, certain strains of *Roultella* and *Klebsiella* have been reported to be capable of growing at temperatures as low as 10°C (Alves et al., 2006; Appel et al., 2021). In the present and previous research (Nan et al., 2022), we observed that panktonic *R. terrigena* grew better at 25°C than 37°C and was also able to grow at 10°C (data not shown). *Raoultella* was the predominant bacterium within biofilms formed at 10°C. There is limited information on the biofilm-forming abilities of *Raoultella*, whereas biofilms are a recognized contributor to the virulence of *Klebsiella pneumoniae* (Seifi et al., 2016). STEC single species strains (T4) did not form or formed weak biofilms at 10°C (*p* < 0.001; Figure 1B).

**Figure 3:**
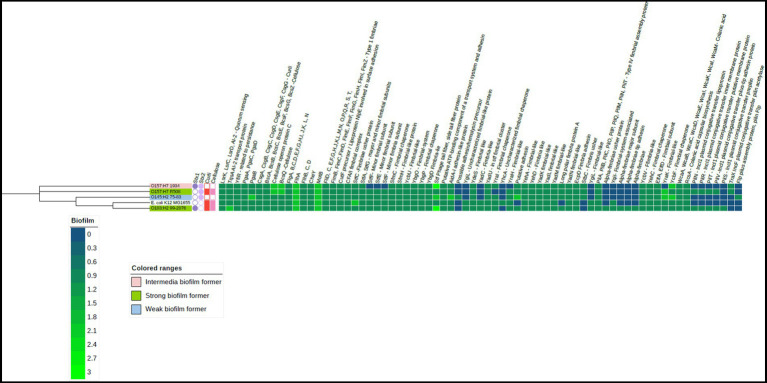
Heat map of the biofilm-related genes in the four Shiga-toxigenic *Escherichia coli* strains. Based on the core genomes of these strains, the phylogenetic tree is displayed alongside with *E. coli* K 12 serving as the reference genome. Gene products corresponding to relevant genes are annotated at the top of the heatmap. Notice that dark blue color means gene absence, dark green indicates one gene copy, and lighter green means more than one gene copy. This visualization also incorporates data on the expression of curli and cellulose genes represented by red and light pink squares, respectively. The presence of Shiga toxin genes is shown by a dark and light purple circles. Figure created using iTOL. V6.

Within the sequenced STEC, the pan-genome encompassed a total of 6,510 distinct protein families. Among these, the core genome consisted of 3,641 protein families that were shared across all the strains. Additionally, each strain exhibited a unique accessory genome. O157 strain 1934 featured 92 specific protein families, R508 had 64, O103 had 380, O145 had 273, and K12 had 393 protein families in their accessory genome. Biofilm formation is a complex process that involves the coordinated expression of genes responsible for initial attachment, maturation, and dispersal of bacteria. The EPS matrix is composed of bacterial appendages such as flagella, pili (fimbriae) and curli, which all have important roles in biofilm formation (Wood et al., 2006; Jin and Marshall, 2020). Our comparative analysis revealed that all STEC strains, along with the generic *E. coli* K12, harbored genes responsible for encoding proteins related to flagella, cellulose synthesis, colonic acid synthesis, quorum sensing, and an array of fimbriae proteins. Some variations were noted in the presence of fimbriae genes (Figure 3). It has been documented that various types of fimbriae play a crucial role in the formation of biofilms (Hancock et al., 2011).

Interestingly, all strains carried genes linked to cellulose production such as *bcs*A, *bcs*B, *bcs*E, *bcs*F, *bcs*G, *bcs*Q, *bcs*Z (Acheson et al., 2021) and genes *pga*A, *pga*B, *pga*C, and *pga*D associated with the production of poly-β-1,6-N-acetyl-D-glucosamine (PNAG) in bacteria. Both cellulose and PNAG are important components of the EPS matrix, as it enhances tolerance to desiccation (Gualdi et al., 2008). Among the STEC strains, only O103 and O157 (R508) produced curli and cellulose at 25°C, which coincided with their ability to form strong biofilms. It is unclear why the 1934 and O145 strains did not exhibit curli or cellulose expression, although the genes were present in their genomes. Additionally, it is unclear why strain 1934 could form an intermediate biofilm compared to O145, which could not. All the STEC strains carried the quorum-sensing genes known to trigger biofilm formation (Preda and Săndulescu, 2019). All the tested STEC strains also harbored the genes responsible for colonic acid production, which confers structural integrity to biofilms (Prigent-Combaret et al., 2000). Plasmids can also play a significant role in biofilm formation as they can carry genes encoding for adhesins, EPS production, and other factors that promote biofilm formation (Cook and Dunny, 2014). The strong biofilm formers, R508 and O103, both possessed IncI-1 plasmids, but it is unclear if they had a role in biofilm formation.

### Bacterial counts in multispecies biofilms

**Figure 4:**
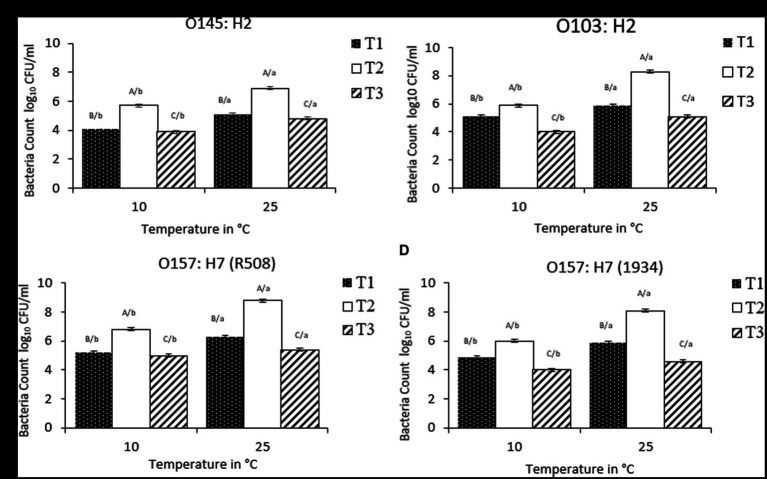
**(A–D)** Number of individual *Escherichia coli* bacteria counts (CFLI/mL) in multispecies biofilms at 10°C and 25^°^C, from T1, T2, and T3, where T1: *Carnobacterium piscicola* + *Lactobacillus bulgaricus* + *E. coli*; T2: Raoultefla sp. + Comamonas sp. + *E. coli*; T3: *Pseudomonas aerugtnosa* + *Comamonas* sp. + *E. coli.* Effects of interaction of treatment (T1, T2, T3) × *E. coli* (1934, O103, O145, R508) x temperature (10°C vs. 25°C; *p* < 0.01). ABCD/, indicates significant differences (*P* ≤ 0.05) between multiple species biofilms treatment within each STEC strain and temperature. /abcd, indicates significant differences (*P* ≤ 0.05) between different temperature within the same multiple biofilm treatment and STEC strain.

Overall, STEC counts within biofilms combinations T1 (*C. piscicola* and *L. bulgaricus*) and T3 (*P. aeruginosa* and *C. korensis*) were lower than STEC counts in T2 (*C. korensis* and *R. terrigena*; Figure 4). The highest STEC reduction was observed in combination with T3 (*Comamonas* sp. and *Pseudomonas*) at 10 and 25°C (*p* < 0.01; Figure 4).

Within T1, at 25°C, STEC counts were as follows: O145:H2 (5.1 log_10_ CFU/mL), O157:H7-R508 (6.3 log_10_ CFU/mL) and for O103 and O157:H7-1934 (5.9 log_10_ CFU/mL). While for T3, at 25°C, the counts were 4.8, 5.1, 5.4 and 4.6 log_10_ CFU/mL for O145, O103, O157-R508 and O157-1934, respectively (*p* < 0.01). At 10°C, a similar response was observed. In contrast, when examining combination T2 (*C. koreensis* + *R. terrigena*), STEC numbers were notably higher, ranging from 6.9 to 8.8 log_10_ CFU/ml, with the lowest count recorded for O145 (Figure 2). Even at 10°C, STEC counts remained elevated within T2, suggesting that the combination of *Raoultella terrigena* and *Comamonas korensis* did not impair the growth of STECs. When looking at STEC controls, single species biofilms, the counts at 10°C were as follows: O145 (5.9 log_10_ CFU/mL), O103 (7.1 log_10_ CFU/mL), O157-R508 (7.8 log_10_ CFU/mL) and O157-1934 (6.4 log_10_ CFU/mL). Meanwhile, at 25°C STEC numbers were as follows: O145 (8 log_10_ CFU/mL), O103 (9 log_10_ CFU/mL), O157-R508 (9 log_10_ CFU/mL), and O157-1934 (8.7 log_10_ CFU/mL; data not shown).

For T1, we observed reductions in STEC at 25°C ranging from 2.7 to 3.1 logs for all four tested strains. To our knowledge there is very limited information on *Carnobacterium* ability to reduce STEC strains which limits our ability to make direct comparisons with our findings. In another hand, *Carnobacterium piscicola* has been reported to produce antimicrobial peptides (Quadri et al., 1994). Some research has reported *Carnobacterium* spp. ability to reduce *Listeria*, with modest reduction of O157:H7 (Danielski et al., 2020). More information is available for other LAB regarding their ability to reduce pathogens. For instance a study aiming to asses a LAB commercial product, composed of a mixture of *Lactobacillus, Lactococcus, and Pediococcus* spp. to reduce STEC on beef strip loins found a modest reduction of 0.4 log10 CFU/cm^2^ (Kirsch et al., 2017). Other research that showed that *Lactobacillus plantarum* and *Lactobacillus rhamnosus*, were able to displace generic *E. coli* pre-established biofilms from medical-grade silicone (Carvalho et al., 2021). Other reports indicated that *Lactobacillus delbrueckii* subsp. *bulgaricus* showed antimicrobial activity against generic *E. coli* (Abedi et al., 2013). Competitive exclusion could perhaps explain this partial inhibition of STEC. Competitive exclusion includes, but is no limited to competition for nutrients, biofilm formation, and production of metabolic substances such as antimicrobial peptides (Abedi et al., 2013). Nevertheless, STEC strains were able to maintain their numbers, with the lowest counts at 4.6 log_10_ CFU/mL at 25°C and 3.9 log10 CFU/mL at 10°C, which is still concerning for food safety (Figure 2).

For LAB and spoilage bacteria, counts varied among biofilm combinations and in some cases at 25°C they were higher than the original inoculum (10^6^ CFU/mL), while they were generally lower at 10°C. *Raoultella* was the exception as it was able to thrive at 10°C.

Synergies among bacteria in mixed biofilms are often strain-rather than species-specific. Various factors, including the composition and the nature of interactions within the biofilm community will influence the development of individual bacterial strains within multi-species biofilms (Elias and Banin, 2012; Ren et al., 2015).

### Biofilm biocide eradication concentration

**Figure 5:**
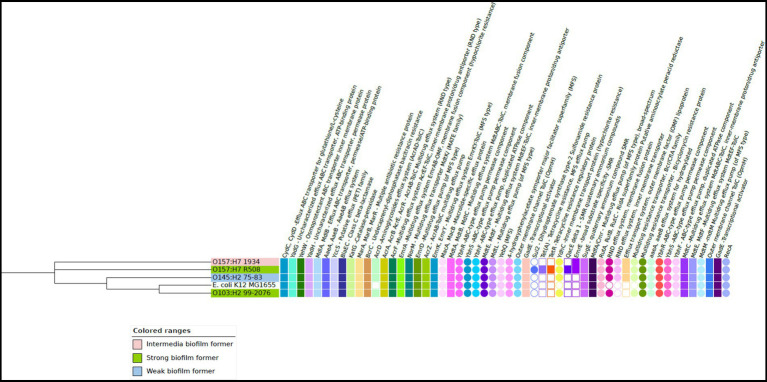
Antimicrobial resistance genes in four STEC strains. Based on the core genomes of these strains, the phylogenetic tree is displayed alongside, with *Escherichia coli* K12 serving as the reference genome. Gene products corresponding to relevant genes are annotated at the top of the binary data set. Solid figures represent gene presence and empty ones, gene absence. Figure created using iTOL. V6.

Biocides were more (*p* < 0.0001) effective against planktonic bacteria than those within biofilms. Furthermore, biocides were more effective at eradicating single-species as compared to multi-species biofilms. At 25°C, all sanitizers, except for sodium hypochlorite, were effective at eliminating STEC biofilms and all were effective at 10°C. We also found that higher concentrations of sodium hypochlorite were needed to eliminate planktonic STEC. At 25°C, O121: H19, O157:H7 (1931), O157:H7 (R508), O145:H7, and O111: NM showed an overall resistance of 15% (*p* < 0.05) to sodium hypochlorite, survival can be found in Table 4. All spoilage and LAB bacteria were sensitive to all biocides, including sodium hypochlorite as they all eradicated biofilms produced by these bacteria at both 10 and 25°C. These findings align with previous reports indicating that planktonic cells are more susceptible to biocides than their biofilm counterparts (Stewart and Costerton, 2001; Levin and Rozen, 2006). It has been reported that EPS within biofilms can inhibit the diffusion of sodium hypochlorite into biofilms and thus limit its ability to contact bacterial cells (De Beer et al., 1994). Furthermore, the efficacy of sodium hypochlorite can be compromised as result of exposure to organic matter within biofilms (Köhler et al., 2018). In the beef industry, it is assumed that organic matter has been adequately cleaned from equipment surfaces before biocides are applied as a failure to do so can compromise their effectiveness (Cramer, 2013). It has been reported that the production of EPS and curli increased the resistance of *E. coli* O157:H7 to chlorine at 22°C. Curli fibers are known to be involved in biofilm formation and can contribute to bacterial resilience by enhancing adhesion to food processing surfaces (Ryu and Beuchat, 2005). The EPS matrices play a significant role in neutralizing or impeding the diffusion of biocides, leading to restricted delivery of their active forms to cells within biofilms (Boyd and Chakrabarty, 1995; Ben-Ari, 1999; Mah, and O'toole, 2001; Ryu and Beuchat, 2005). Interestingly all four STEC possessed curli genes, however strains O145 and O157 (1934) did not synthetize curli or cellulose (phenotype) when tested *in-vitro* on Congo red and LB agar supplemented with calcofluor white (Nan et al., 2022). Whole genome sequencing revealed that strains O121:H19, O157:H7 (1931), O157:H7 (R508), O45:H7, and O111:NM did carry genes coding for *acr*AB-TolC, *emr*AB-TolC and *ydh*C which have been linked to resistance to biocides (Figure 5; Piddock, 2006; Wand et al., 2022).

**Table 4:** The sanitizer resistance of the STEC in planktonic and sessile stages (single-species biofilm) against regular sanitizers often used in food processing facilities at 25°C and 10°C.

STEC Strain	PAA			Quats (GM)			HyP			Quats (PQ)			Shyd			Shypo			Planktonic	Sessile		Planktonic	Sessile		Planktonic	Sessile		Planktonic	Sessile		Planktonic	Sessile		Planktonic	Sessile	
**Temperature**	**25°C**	**25°C**	**10°C**	**25°C**	**25°C**	**10°C**	**25°C**	**25°C**	**10°C**	**25°C**	**25°C**	**10°C**	**25°C**	**25°C**	**10°C**	**25°C**	**25°C**	**10°C**
*O145: H2*	S	S	S	S	S	S	S	S	S	S	S	S	S	S	S	S	S	S
*O121: H19*	S	S	S	S	S	S	S	S	S	S	S	S	S	S	S	S	**R**	S
*O157:H7 1934*	S	S	S	S	S	S	S	S	S	S	S	S	S	S	S	S	S	S
*O157:H7 1931*	S	S	S	S	S	S	S	S	S	S	S	S	S	S	S	S	**R**	S
*O26: H11*	S	S	S	S	S	S	S	S	S	S	S	S	S	S	S	S	S	S
*O157:H7 R508*	S	S	S	S	S	S	S	S	S	S	S	S	S	S	S	S	**R**	S
*O45:H7*	S	S	S	S	S	S	S	S	S	S	S	S	S	S	S	S	**R**	S
*O111:NM*	S	S	S	S	S	S	S	S	S	S	S	S	S	S	S	S	**R**	S
*O103:H2*	S	S	S	S	S	S	S	S	S	S	S	S	S	S	S	S	S	S

An analytical study was conducted to determine the susceptibility and resistance of various STEC strains to different sanitizers. The calculation method utilized the recommended threshold values outlined by the manufacturer, which were as follows: Organic peroxy acid (PAA) = 600 ppm, Sodium hydroxide (Shyd) = 2,500 ppm, Sodium hypochlorite (Shypo) = 1,200 ppm, Quats (Germarc) = 600 ppm, Hydrogen peroxide (HyP) = 250 ppm, Quats (Powerquat) = 550 ppm. The investigation included both planktonic bacteria and the sessile stage of a single species. The results were categorized as “S” for susceptibility and “R” for resistance, respectively.

The low susceptibility of STEC biofilms to sodium hypochlorite is complex and may be attributed to the different factors that could be playing a role, such as a biofilm matrix, chlorine concentrations, presence of organic matter, presence of other bacteria and efflux pumps that may accelerate the clearance of this biocide from the cell. These findings highlight the importance of implementing effective cleaning and sanitation procedures to prevent the formation and persistence of biofilms in food processing environments (Yuan et al., 2022).

### Multispecies biofilms response to biocides

The extent of STEC resilience was influenced by temperature, the specific combination of bacterial strains within the biofilm, and the STEC strain involved (Table 5).

**Table 5:** The percentage of STECs survival (*P* < 0.01) in multispecies biofilm against regular biocides often used in food processing facilities [quats, sodium hypochlorite (Shypo), sodium hydroxide (Shyd), hydrogen peroxide (HyP), peroxyacetic acid (PAA)] at 10°C and 25°C temperature on day 6.

Multispecies	PAA (*n* = 0)		Shyd (*n* = 20)		Shypo (*n* = 20)		Quats (GM) (*n* = 19)		HyP (*n* = 27)		Quats (PQ) (*n* = 18)		10°C	25°C	10°C	25°C	10°C	25°C	10°C	25°C	10°C	25°C	10°C	25°C
*Carnobacterium piscicola* + *Lactobacillus Bulgaricus*, *n* (%)												
O157:H7(1934)	0	0	0	15	0	0	0	11.11	0	0	0	0
O103:H2	0	0	0	30	0	0	0	22.22	0	16.67	0	0
O145:H2	0	0	0	0	0	10.53	0	11.11	0	0	0	0
O157:H7(R508)	0	0	0	0	0	26.32	0	11.11	0	0	0	0
T4	0	0	0	0	0	0	0	0	0	0	0	0
*Raoultella* sp. + *Comamonas* sp., *n* (%)												
O157:H7(1934)	0	0	0	0	0	0	0	11.11	0	0	0	16.67
O103:H2	0	0	0	30	0	15.79	0	22.22	0	0	16.67	0
O145:H2	0	0	0	0	0	0	0	0	0	0	0	0
O157:H7(R508)	0	0	0	25	0	15.79	0	11.11	16.67	16.67	0	0
T4	0	0	0	11.11	0	0	0	0	0	0	0	0
*Pseudomonas aeruginosa* + *Comamonas* sp., *n* (%)												
O157:H7(1934)	0	0	0	0	0	0	0	0	0	0	16.67	0
O103:H2	0	0	0	0	0	31.58	0	0	16.67	0	16.67	0
O145:H2	0	0	0	0	0	0	0	0	0	0	0	0
O157:H7(R508)	0	0	0	20	0	25	0	0	0	16.67	33.33	0
T4	0	0	0	0	0	11.11	0	0	0	0	0	0

x Chi-square analysis indicated the *E. coli* disinfectant resistance was different by sanitizer and multispecies group (*P* < 0.01). The proportion of resistant sessile strain to each biocide was calculated using a threshold value given by the manufacturer (Shypo = 1,200 ppm; Shyd = 2,500 ppm; Quats [PQ] = 550 ppm; Shyd = 250 ppm; Quats[GM] = 600 ppm; PAA = 600 ppm) T1: *Carnobacterium piscicola* + *Lactobacillus Bulgaricus;* T2: *Raoultella* sp. + *Comamonas* sp.; T3: *Pseudomonas aeruginosa* + *Comamonas* sp. T4: survival percentage of multi-species spoilage bacteria without *Escherichia coli*.

At 10°C, T1 consisting of *Carnobacterium piscicola* and *Lactobacillus bulgaricus*, resulted in a 100% reduction of STEC when treated with all sanitizers. In contrast, for T2 (*Comamonas koreensis + Raoultella terrigena*), O157:H7 (strain R508) had a survival rate of 16.7% after treatment with 3rd generation Quats (PQ). In comparison, O103 showed a similar survival rate of 16.7% after exposure to hydrogen peroxide.

Interestingly, in biofilm combination T3 at 10°C, we observed that STEC survival was notably higher following exposure to 3rd generation Quaternary Ammonium Compounds (QACs) and hydrogen peroxide. The reasons for this phenomenon are not entirely clear at this stage. It is possible that stress responses or the activation of genes encoding efflux pumps could be contributing factors. However, it’s worth noting that at a lower temperature like 10°C, one might expect a slowdown in physiological responses. On the other hand, the key differentiating factor appears to be the specific bacterial composition within the biofilm. It is plausible that the presence of both *Pseudomonas* and *Commamonas* in the biofilm could influence STEC survival. This needs more research.

*Escherichia coli* O157:H7 1934, which is an intermediate biofilm former and O103, a strong biofilm former, showed a survival rate of 16.7%. However, the strong biofilm former, R508 had an even higher survival rate of 33.3% when exposed to Quats (PQ) whereas O103 had a survival rate of 16.7% when exposed to hydrogen peroxide.

Multispecies biofilms formed at 25°C exhibited greater resilience compared to those formed at 10°C, likely due to greater development of the EPS matrix at 25°C (Figure 1). For biofilm combination T1, all STEC strains were eliminated (100%) when treated with Biodestroy, a peroxy acid-based biocide specifically developed to eradicate biofilms. However, when exposed to other sanitizers, some STEC strains survived. Overall, we observed varying levels of survival among the tested disinfectants. Quaternary ammonium compounds (GM) resulted in the highest survival rates, followed by sodium hypochlorite, power quat, and hydrogen peroxide (Table 5). All STEC strains survived exposure to first-generation Quats in lactic acid biofilm T1 at 25°C. In biofilm T2, only O145 was eliminated, while in biofilm T3, all STEC strains were effectively eliminated (Table 5). Notably, *E. coli* O145, a weak biofilm former, survived exposure to sodium hypochlorite and first-generation Quats within lactic acid bacteria biofilms (T1), but was eliminated by biocides in T2 and T3 biofilms. When exposed to biocides STEC survival within single-species biofilms was lower than the survival observed within multispecies biofilms. Within single species biofilms, only O103:H2 and O157 (R508) strains managed to survive after treatment with sodium hydroxide (15.8%) and sodium hypochlorite (66.7%).

Interestingly, R508 remained more viable after exposure to sodium hypochlorite in single species biofilms (66.7%) than in multi-species biofilms. Bacteria within multispecies biofilms responded differently to biocide exposure. The EPS matrix serves as a shield that protects bacteria against environmental stresses such as antimicrobial substances. The EPS matrix density can be affected by temperature as well as by the type and number of bacterial species within the biofilm, which also impact EPS production. Temperature is also likely affecting bacteria’s metabolic activity and their ability to respond to environmental stress. As expected, the type of biocide also affects STEC’s ability to survive (Levin and Rozen, 2006; Surdeau et al., 2006).

In this study, we analyzed the genomes of the four STEC strains to identify the presence of genes associated with resistance to biocides. A total of 62 genes coding for proteins related to antimicrobial resistance were identified (Figure 5), with some of these implicated in biocide resistance. For example, *AcrAB-TolC, EmrAB-TolC* and *YdhC* have been associated with resistance to sodium hypochlorite and *katG* has been linked to resistance to hydrogen peroxide (Sun et al., 2016; Loewen et al., 2018). Other genes related to quaternary ammonium resistance were also found, such as *qac*E delta 1, *sug*E and *yeh*W (Castro et al., 2023).

Among all the *E. coli* strains studied O157:H7 strain R508 was the only one to carry all 62 resistance genes. In contrast, strain 1934 lacked the *tetA, tetR, qacE delta-1,* and *sul2* genes. *E. coli* O145 lacked *tetA, tetR, qacE delta-1,* and *emrE* genes, while *E. coli* O103 was deficient in *sul*2, *tet*A, *tet*R, *qac*E delta-1, *emr*E, the RND efflux system, and the efflux transport system OMF. The generic strain, *E. coli* K12 was found to lack 11 of the 62 identified resistance genes.

Given the variations in gene compositions, one might have anticipated that strain R508 would demonstrate reduced susceptibility to biocides. However, upon scrutinizing the Minimum Bactericidal concentration and survival rates across these strains, no significant differences were discerned. More research is needed to determine whether differences in gene expression could help explain these findings.

### Spoilage survival in multispecies biofilms

Spoilage bacteria and LAB bacteria exhibited a greater sensitivity to biocides than STEC strains. Combination T2 and T3 showed in general, a higher bacterial survival compared to T1 (Table 6). Similar to previous observations on STEC, the survival of SP and LAB bacteria was influenced by temperature. At 10°C, biofilms were eliminated by Biodestroy. However, some level of survival was observed when these biofilms were exposed to sodium hydroxide (ranging from 0.4% to 5.4%), Quats (GM: 4% to 8%; PQ: 3.4% to 6.8%) and hydrogen peroxide (5.5% to 11%; Table 6).

**Table 6:** The percentage of survival of spoilage bacteria in multispecies biofilm associated with STECs against regular biocides often used in food processing facilities [quats, sodium hypochlorite (Shypo), sodium hydroxide (Shyd), hydrogen peroxide (HyP), peroxyacetic acid (PAA)] at 10°C and 25°C temperature on day 6.

Multispecies	PAA (*n* = 0)		Shyd (*n* = 20)		Shypo (*n* = 15)		Quats(GM) (*n* = 21)		HyP (*n* = 15)		Quats (PQ) (*n* = 15)		10°C	25°C	10°C	25°C	10°C	25°C	10°C	25°C	10°C	25°C	10°C	25°C
T1, T2, T3 with *Escherichia coli* O157: H7 (1934) *n* (%)												
T1	0	0	2.70	3.60	0	9.38	0	0	0	0	0	3.41
T2	0	0	2.70	4.50	0	0	0	8.11	0	0	0	6.82
T3	0	0	0.41	4.50	0	15.63	4.05	6.76	5.56	11.11	6.82	6.82
T4	0	0	0	0	0	0	0	0	0	0	0	0
T1, T2, T3 with *E. coli* O103: H2 *n* (%)												
T1	0	0	5.41	5.41	0	0	0	4.05	0	5.56	0	3.41
T2	0	0	2.70	5.41	0	9.38	0	8.11	5.56	0	3.41	6.82
T3	0	66.67	5.41	4.50	0	18.75	4.05	8.11	5.56	11.11	6.82	6.82
T4	0	0	0	4.50	0	0	0	0	0	0	0	0
T1, T2, T3 with *E. coli* O145: H2 *n* (%)												
T1	0	0	5.41	3.60	0	0	0	5.41	0	0	0	2.27
T2	0	0	2.70	5.41	0	12.50	4.05	8.11	0	5.56	0	6.82
T3	0	0	5.41	2.70	0	0	4.05	6.76	0	11.11	6.82	5.68
T4	0	0	0	0	0	0	0	0	0	0	0	0
T1, T2, T3 with *E. coli* O157: H7 (R508) *n* (%)												
T1	0	0	2.70	4.50	0	12.50	0	4.05	0	5.56	0	6.82
T2	0	33.33	0	5.41	0	15.63	0	8.11	0	5.56	0	6.82
T3	0	0	5.41	4.50	0	6.25	8.11	8.11	11.11	11.11	6.82	6.82
T4	0	0	0	0	0	33.33	0	0	0	0	0	0

x Chi-square analysis indicated the biocide resistance of spoilage bacteria differed with biocide and multispecies group (*P* < 0.01). The proportion of resistant sessile strains to each biocide was calculated using a threshold value provided by the manufacturer (Shypo = 1,200 ppm; Shyd = 2,500 ppm; Quats [PQ] = 550 ppm; Shyd = 250 ppm; Quats [GM] = 600 ppm; PAA = 600 ppm) T1: *Carnobacterium. Piscicola* + *Lactobacillus Bulgaricus;* T2: *Raoultella* sp. + *Comamonas* sp.; T3: *Pseudomonas aeruginosa* + *Comamonas* sp. T4: Positive Control used to estimate the precent survival of a single species of *E. coli*.

At 25°C, higher SP and LAB survival rates were observed in biofilm combinations T3 (66.7%) and T2 (33.3%) after treatment with Biodestroy (Table 6). The survival rates for sodium hydroxide ranged from 3.5% to 5.4%, from 6.25% to 18.8% for sodium hypochlorite, and from 5.5% to 11% for hydrogen peroxide. From 4 to 8% for Quats (GM), and from 2% to 6.8% for Quats (PQ). Interestingly, when STEC were not included in the biofilm (control—T4), the survival rates of LAB and spoilage bacteria were dramatically lower (Table 6).

Overall, spoilage within combination T2 appeared to exhibit higher resistance to all tested biocides than other combinations.

It is important to highlight that the overall survival of spoilage microorganisms was notably higher in multispecies biofilms that included STEC strains. When examining controls comprised exclusively of multispecies biofilms without any STEC strains, the survival of spoilage microorganisms was much lower than the survival rates observed in multispecies biofilms plus STEC biofilms formed at 25°C. Specifically, in the case of combination T2 (*Raoultella* and *Comamonas*), the survival rate was 22.2% when exposed to sodium hypochlorite. The survival rate for combination T3 (*Raoultella* and *Pseudomonas*) when treated with sodium hydroxide was 11.1%. It is not clear how the participation of STEC in the biofilms enhanced the ability of spoilage and LAB’s to survive. Perhaps the contribution of extracellular polymers to the biofilm by STEC further enhanced the barrier responsible for decreasing the effectiveness of biocides.

Gram-negative bacteria like STEC exhibit greater resistance to many antiseptics and disinfectants compared to Gram-positive bacteria (McDonnell and Russell, 1999; Russell, 1999; Wickham, 2017; Breijyeh et al., 2020). This resistance is linked to differences in lipopolysaccharide (LPS) composition and cation content of the outer membrane in Gram-negative bacteria, particularly their high magnesium ion (Mg2+) content, which promotes strong LPS–LPS interactions. Furthermore, the small size of porins in Gram-negative bacteria can restrict the diffusion of certain molecules through the membrane. The LPS of Gram-negative bacteria typically contains phosphate-linked arabinose, which reduces the outer membrane’s affinity for certain antibiotics and positively charged molecules (McDonnell and Russell, 1999). In addition, the presence of efflux pumps acting alone or in combination with porins is increasingly recognized as an essential permeability barrier (Piddock, 2006), likely aiding Gram-negative increased resistance. Overall, the extent of STEC susceptibility to biocides varied depending on factors such as temperature, the specific combination of bacterial strains within the biofilm, biocide type and the characteristics of the STEC strain involved.

## Data availability statement

The datasets presented in the study have been deposited to the Dryad Repository (https://datadryad.org/stash), under doi: 10.5061/dryad.zpc866tgh.

## Author contributions

KK: Methodology, Writing – original draft, Formal analysis. AR-G: Formal analysis, Writing – review & editing. CN: Writing – review & editing, Conceptualization, Methodology. TM: Conceptualization, Methodology, Writing – review & editing. XY: Conceptualization, Writing – review & editing. CN-B: Conceptualization, Writing – review & editing, Data curation, Funding acquisition, Investigation, Methodology, Resources, Software, Supervision, Visualization, Writing – original draft.

## Footnotes

^1^ https://www.bv-brc.org/

## References

[ref1] Abedi D. Feizizadeh S. Akbari V. Jafarian-Dehkordi A. (2013). In vitro anti-bacterial and anti-adherence effects of *Lactobacillus delbrueckii* subsp bulgaricus on *Escherichia coli*. Res Pharm Sci 8, 260–268. PMID: 24082895 PMC3757591

[ref2] Acheson J. F. Ho R. Goularte N. F. Cegelski L. Zimmer J. (2021). Molecular organization of the *E. coli* cellulose synthase macrocomplex. Nat. Struct. Mol. Biol. 28, 310–318. doi: 10.1038/s41594-021-00569-7, PMID: 33712813 PMC9278871

[ref9001] Adator E. H. Cheng M. Holley R. Mcallister T. Narvaez-Bravo C. (2018). Ability of Shiga toxigenic Escherichia coli to survive within dry-surface biofilms and transfer to fresh lettuce. Int J Food Microbiol 269, 52–59. 29421358 10.1016/j.ijfoodmicro.2018.01.014

[ref3] Alves M. S. Dias R. C. De Castro A. C. Riley L. W. Moreira B. M. (2006). Identification of clinical isolates of indole-positive and indole-negative klebsiella spp. J. Clin. Microbiol. 44, 3640–3646. doi: 10.1128/JCM.00940-06, PMID: 16928968 PMC1594763

[ref5] Appel T. M. Quijano-Martínez N. De La Cadena E. Mojica M. F. Villegas M. V. (2021). Microbiological and clinical aspects of Raoultella spp. Front. Public Health 9:686789. doi: 10.3389/fpubh.2021.686789, PMID: 34409007 PMC8365188

[ref6] Araújo P. A. (2014). Biofilm control with antimicrobial agents: The role of the exopolymeric matrix. University of Porto. Available at: https://core.ac.uk/download/pdf/143412979.pdf

[ref7] Beier R. C. Franz E. Bono J. L. Mandrell R. E. Fratamico P. M. Callaway T. R.. (2016). Disinfectant and antimicrobial susceptibility profiles of the big six non-O157 Shiga toxin–producing *Escherichia coli* strains from food animals and humans. J. Food Prot. 79, 1355–1370. doi: 10.4315/0362-028X.JFP-15-600, PMID: 27497123

[ref8] Ben-Ari E. T. (1999). Not just slime: beneath the slippery exterior of a microbial bio film lies a remarkably organized community of organisms. Bioscience 49, 689–695. doi: 10.2307/1313592

[ref9] Bloomfield S. F. Uso E. E. (1985). The antibacterial properties of sodium hypochlorite and sodium dichloroisocyanurate as hospital disinfectants. J. Hosp. Infect. 6, 20–30. doi: 10.1016/S0195-6701(85)80014-1, PMID: 2859319

[ref10] Boyd A. Chakrabarty A. M. (1995). *Pseudomonas aeruginosa* biofilms: role of the alginate exopolysaccharide. J. Ind. Microbiol. 15, 162–168. doi: 10.1007/BF01569821 8519473

[ref11] Breijyeh Z. Jubeh B. Karaman R. (2020). Resistance of gram-negative bacteria to current antibacterial agents and approaches to resolve it. Molecules 25:1340. doi: 10.3390/molecules25061340, PMID: 32187986 PMC7144564

[ref12] Bridges D. F. Lacombe A. Wu V. C. H. (2022). Fundamental differences in inactivation mechanisms of *Escherichia coli* O157:H7 between chlorine dioxide and sodium hypochlorite. Front. Microbiol. 13:923964. doi: 10.3389/fmicb.2022.923964, PMID: 35783445 PMC9247566

[ref13] Canada G. O. (2016). National enteric surveillance program annual summary 2014. Public Health Agency of Canada Guelph, Ontario.

[ref14] Carvalho F. M. Mergulhão F. J. M. Gomes L. C. (2021). Using lactobacilli to fight Escherichia coli and *Staphylococcus aureus* biofilms on urinary tract devices. Antibiotics 10:1525. doi: 10.3390/antibiotics10121525 34943738 PMC8698619

[ref15] Casaburi A. Nasi A. Ferrocino I. Di Monaco R. Mauriello G. Villani F.. (2011). Spoilage-related activity of *Carnobacterium maltaromaticum* strains in air-stored and vacuum-packed meat. Appl. Environ. Microbiol. 77, 7382–7393. doi: 10.1128/AEM.05304-11, PMID: 21784913 PMC3194841

[ref16] Castro V. S. Conte C. A. Jr. De Souza Figueiredo E. E. Yang X. Stanford K. (2023). Efficacy of quaternary ammonium compounds for control of individual and mixed cultures of *Escherichia coli* with high- and low-quaternary ammonium compounds resistance. Foodborne Pathog. Dis. 20, 261–269. doi: 10.1089/fpd.2023.0005, PMID: 37379475

[ref17] CDC (2023). Shiga toxin-producing *E. coli* and Leafy Greens. Available at: https://www.cdc.gov/ncezid/dfwed/prevention-priorities/ecoli-and-leafy-greens.html.

[ref18] Chapman J. S. (2003). Disinfectant resistance mechanisms, cross-resistance, and co-resistance. Int. Biodeter. Biodegr. 51, 271–276. doi: 10.1016/S0964-8305(03)00044-1

[ref19] Cook L. C. Dunny G. M. (2014). The influence of biofilms in the biology of plasmids. Microbiol Spectr 2:0012. PMID: 25392747 10.1128/microbiolspec.PLAS-0012-2013 PMC4225719

[ref20] Cramer M. M. (2013). Food plant sanitation: Design, maintenance, and good manufacturing practices. 2nd Edn. Boca Raton: CRC Press.

[ref21] Danielski G. M. Imazaki P. H. Andrade Cavalari C. M. Daube G. Clinquart A. Macedo R. E. F. (2020). *Carnobacterium maltaromaticum* as bioprotective culture in vitro and in cooked ham. Meat Sci. 162:108035. doi: 10.1016/j.meatsci.2019.108035, PMID: 31855662

[ref22] De Beer D. Srinivasan R. Stewart P. S. (1994). Direct measurement of chlorine penetration into biofilms during disinfection. Appl. Environ. Microbiol. 60, 4339–4344. doi: 10.1128/aem.60.12.4339-4344.1994, PMID: 7811074 PMC201990

[ref23] Elias S. Banin E. (2012). Multi-species biofilms: living with friendly neighbors. FEMS Microbiol. Rev. 36, 990–1004. doi: 10.1111/j.1574-6976.2012.00325.x, PMID: 22229800

[ref24] Fouladkhah A. Geornaras I. Sofos J. N. (2013). Biofilm formation of O157 and non-O157 Shiga toxin-producing Escherichia coli and multidrug-resistant and susceptible salmonella typhimurium and Newport and their inactivation by sanitizers. J. Food Sci. 78, M880–M886. doi: 10.1111/1750-3841.12123, PMID: 23601046

[ref25] Gualdi L. Tagliabue L. Bertagnoli S. Ieranò T. De Castro C. Landini P. (2008). Cellulose modulates biofilm formation by counteracting curli-mediated colonization of solid surfaces in *Escherichia coli*. Microbiology 154, 2017–2024. doi: 10.1099/mic.0.2008/018093-0, PMID: 18599830

[ref26] Hancock V. Witsø I. L. Klemm P. (2011). Biofilm formation as a function of adhesin, growth medium, substratum and strain type. Int. J. Med. Microbiol. 301, 570–576. doi: 10.1016/j.ijmm.2011.04.018, PMID: 21646046

[ref27] Jin X. Marshall J. S. (2020). Mechanics of biofilms formed of bacteria with fimbriae appendages. PLoS One 15:e0243280. doi: 10.1371/journal.pone.0243280, PMID: 33290393 PMC7723297

[ref28] Kirsch K. R. Tolen T. N. Hudson J. C. Castillo A. Griffin D. Taylor T. M. (2017). Effectiveness of a commercial lactic acid bacteria intervention applied to inhibit Shiga toxin-producing *Escherichia coli* on refrigerated vacuum-aged beef. Int. J. Food Sci. 2017:8070515. doi: 10.1155/2017/8070515 28630857 PMC5463119

[ref29] Köhler A. T. Rodloff A. C. Labahn M. Reinhardt M. Truyen U. Speck S. (2018). Efficacy of sodium hypochlorite against multidrug-resistant gram-negative bacteria. J. Hosp. Infect. 100, e40–e46. doi: 10.1016/j.jhin.2018.07.017 30026008

[ref30] Lambert R. J. Pearson J. (2000). Susceptibility testing: accurate and reproducible minimum inhibitory concentration (mic) and non-inhibitory concentration (Nic) values. J. Appl. Microbiol. 88, 784–790. doi: 10.1046/j.1365-2672.2000.01017.x 10792538

[ref31] Levin B. R. Rozen D. E. (2006). Non-inherited antibiotic resistance. Nat. Rev. Microbiol. 4, 556–562. doi: 10.1038/nrmicro1445, PMID: 16778840

[ref32] Loewen P. C. De Silva P. M. Donald L. J. Switala J. Villanueva J. Fita I.. (2018). KatG-mediated oxidation leading to reduced susceptibility of bacteria to kanamycin. ACS Omega 3, 4213–4219. doi: 10.1021/acsomega.8b00356, PMID: 29732452 PMC5928485

[ref33] Ma Z. Bumunang E. W. Stanford K. Bie X. Niu Y. D. Mcallister T. A. (2019). Biofilm formation by Shiga toxin-producing *Escherichia coli* on stainless steel coupons as affected by temperature and incubation time. Microorganisms 7:95. doi: 10.3390/microorganisms7040095, PMID: 30935149 PMC6518284

[ref34] Mah T.-F. C. O'toole G. A. (2001). Mechanisms of biofilm resistance to antimicrobial agents. Trends Microbiol. 9, 34–39. doi: 10.1016/S0966-842X(00)01913-2 11166241

[ref35] Marouani-Gadri N. Augier G. Carpentier B. (2009). Characterization of bacterial strains isolated from a beef-processing plant following cleaning and disinfection—influence of isolated strains on biofilm formation by Sakaï and Edl 933 *E. coli* O157:H7. Int. J. Food Microbiol. 133, 62–67. doi: 10.1016/j.ijfoodmicro.2009.04.028, PMID: 19446903

[ref36] Mcdonnell G. Russell A. D. (1999). Antiseptics and disinfectants: activity, action, and resistance. Clin. Microbiol. Rev. 12, 147–179. doi: 10.1128/CMR.12.1.147, PMID: 9880479 PMC88911

[ref37] Melton-Celsa A. R. Robinson C. M. Smith M. J. (2007). Shiga Toxins (Stxs): Multifaceted Pathogenicity Determinants. 239–251. doi: 10.1128/9781555815851.ch16

[ref38] Nan Y. Rodas-Gonzalez A. Stanford K. Nadon C. Yang X. Mcallister T.. (2022). Formation and transfer of multi-species biofilms Containing *E. coli* O103:H2 on food contact surfaces to beef. Front. Microbiol. 13:863778. doi: 10.3389/fmicb.2022.863778, PMID: 35711784 PMC9196126

[ref9002] Overbeek R. Begley T. Butler R. M. Choudhuri J. V. Chuang H. Y. Cohoon M.. (2005). The subsystems approach to genome annotation and its use in the project to annotate 1000 genomes. Nucleic Acids Res 33, 5691–5702. 16214803 10.1093/nar/gki866 PMC1251668

[ref9003] Overbeek R. Olson R. Pusch G. D. Olsen G. J. Davis J. J. Disz T.. (2014). The SEED and the Rapid Annotation of microbial genomes using Subsystems Technology (RAST). Nucleic Acids Res 42, D206–214. 24293654 10.1093/nar/gkt1226 PMC3965101

[ref39] Perez R. H. Zendo T. Sonomoto K. (2014). Novel bacteriocins from lactic acid bacteria (lab): various structures and applications. Microb. Cell Fact. 13:S3. doi: 10.1186/1475-2859-13-S1-S3, PMID: 25186038 PMC4155820

[ref40] Piddock L. J. (2006). Clinically relevant chromosomally encoded multidrug resistance efflux pumps in bacteria. Clin. Microbiol. Rev. 19, 382–402. doi: 10.1128/CMR.19.2.382-402.2006, PMID: 16614254 PMC1471989

[ref41] Poimenidou S. V. Chrysadakou M. Tzakoniati A. Bikouli V. C. Nychas G. J. Skandamis P. N. (2016). Variability of *Listeria monocytogenes* strains in biofilm formation on stainless steel and polystyrene materials and resistance to peracetic acid and quaternary ammonium compounds. Int. J. Food Microbiol. 237, 164–171. doi: 10.1016/j.ijfoodmicro.2016.08.029, PMID: 27585076

[ref42] Preda V. G. Săndulescu O. (2019). Communication is the key: biofilms, quorum sensing, formation and prevention. Discoveries 7:e100. doi: 10.15190/d.2019.13 32309618 PMC7086079

[ref43] Prigent-Combaret C. Prensier G. Le Thi T. T. Vidal O. Lejeune P. Dorel C. (2000). Developmental pathway for biofilm formation in curli-producing *Escherichia coli* strains: role of flagella, curli and colanic acid. Environ. Microbiol. 2, 450–464. doi: 10.1046/j.1462-2920.2000.00128.x, PMID: 11234933

[ref44] Quadri L. E. Sailer M. Roy K. L. Vederas J. C. Stiles M. E. (1994). Chemical and genetic characterization of bacteriocins produced by *Carnobacterium piscicola* Lv17B. J. Biol. Chem. 269, 12204–12211. doi: 10.1016/S0021-9258(17)32702-3, PMID: 8163526

[ref45] Quintieri L. Fanelli F. Caputo L. (2019). Antibiotic resistant pseudomonas Spp. spoilers in fresh dairy products: an underestimated risk and the control strategies. Food Secur. 8:372. doi: 10.3390/foods8090372, PMID: 31480507 PMC6769999

[ref46] Ren D. Madsen J. S. Sørensen S. J. Burmølle M. (2015). High prevalence of biofilm synergy among bacterial soil isolates in cocultures indicates bacterial interspecific cooperation. ISME J. 9, 81–89. doi: 10.1038/ismej.2014.96, PMID: 24936766 PMC4274433

[ref47] Rodríguez-Melcón C. Alonso-Calleja C. García-Fernández C. Carballo J. Capita R. (2021). Minimum inhibitory concentration (mic) and minimum bactericidal concentration (Mbc) for twelve antimicrobials (biocides and antibiotics) in eight strains of *Listeria monocytogenes*. Biology 11:46. doi: 10.3390/biology11010046 35053044 PMC8773323

[ref48] Russell A. D. (1999). Bacterial resistance to disinfectants: present knowledge and future problems. J. Hosp. Infect. 43, S57–S68. doi: 10.1016/S0195-6701(99)90066-X, PMID: 10658759

[ref49] Ryu J. H. Beuchat L. R. (2005). Biofilm formation by *Escherichia coli* O157:H7 on stainless steel: effect of exopolysaccharide and curli production on its resistance to chlorine. Appl. Environ. Microbiol. 71, 247–254. doi: 10.1128/AEM.71.1.247-254.2005, PMID: 15640194 PMC544232

[ref50] Sagripanti J. L. Bonifacino A. (2000). Resistance of *Pseudomonas aeruginosa* to liquid disinfectants on contaminated surfaces before formation of biofilms. J. AOAC Int. 83, 1415–1422. doi: 10.1093/jaoac/83.6.1415, PMID: 11128146

[ref51] Seifi K. Kazemian H. Heidari H. Rezagholizadeh F. Saee Y. Shirvani F.. (2016). Evaluation of biofilm formation among *Klebsiella pneumoniae* isolates and molecular characterization by Eric-Pcr. Jundishapur J Microbiol 9:e30682. doi: 10.5812/jjm.30682 27099694 PMC4834130

[ref52] Sidhu M. S. Langsrud S. Holck A. (2001). Disinfectant and antibiotic resistance of lactic acid bacteria isolated from the food industry. Microb. Drug Resist. 7, 73–83. doi: 10.1089/107662901750152846, PMID: 11310806

[ref53] Silagyi K. Kim S. H. Lo Y. M. Wei C. I. (2009). Production of biofilm and quorum sensing by *Escherichia coli* O157:H7 and its transfer from contact surfaces to meat, poultry, ready-to-eat deli, and produce products. Food Microbiol. 26, 514–519. doi: 10.1016/j.fm.2009.03.004, PMID: 19465248

[ref54] Smith J. L. Fratamico P. M. Gunther N. W. T. (2014). Shiga toxin-producing *Escherichia coli*. Adv. Appl. Microbiol. 86, 145–197. doi: 10.1016/B978-0-12-800262-9.00003-2 24377855

[ref55] Stewart P. S. Costerton J. W. (2001). Antibiotic resistance of bacteria in biofilms. Lancet 358, 135–138. doi: 10.1016/S0140-6736(01)05321-1 11463434

[ref56] Sun D. Crowell S. A. Harding C. M. De Silva P. M. Harrison A. Fernando D. M.. (2016). KatG and KatE confer Acinetobacter resistance to hydrogen peroxide but sensitize bacteria to killing by phagocytic respiratory burst. Life Sci. 148, 31–40. doi: 10.1016/j.lfs.2016.02.015, PMID: 26860891 PMC4792659

[ref57] Surdeau N. Laurent-Maquin D. Bouthors S. Gellé M. P. (2006). Sensitivity of bacterial biofilms and planktonic cells to a new antimicrobial agent, Oxsil® 320N. J. Hosp. Infect. 62, 487–493. doi: 10.1016/j.jhin.2005.09.003, PMID: 16478644

[ref58] Thi M. T. T. Wibowo D. Rehm B. H. A. (2020). *Pseudomonas aeruginosa* Biofilms. Int. J. Mol. Sci. 21:8671. doi: 10.3390/ijms21228671, PMID: 33212950 PMC7698413

[ref59] Visvalingam J. Wang H. Ells T. C. Yang X. (2019). Facultative anaerobes shape multispecies biofilms composed of meat processing surface bacteria and *Escherichia coli* O157:H7 or *Salmonella enterica* serovar typhimurium. Appl. Environ. Microbiol. 85:19. doi: 10.1128/AEM.01123-19, PMID: 31253683 PMC6696955

[ref60] Vogeleer P. Tremblay Y. D. Mafu A. A. Jacques M. Harel J. (2014). Life on the outside: role of biofilms in environmental persistence of Shiga-toxin producing *Escherichia coli*. Front. Microbiol. 5:317. doi: 10.3389/fmicb.2014.00317 25071733 PMC4076661

[ref61] Wand M. E. Darby E. M. Blair J. M. A. Sutton J. M. (2022). Contribution of the efflux pump Acrab-TolC to the tolerance of chlorhexidine and other biocides in klebsiella spp. J. Med. Microbiol. 71:1496. doi: 10.1099/jmm.0.001496, PMID: 35324422 PMC9176267

[ref62] Wang R. Bono J. L. Kalchayanand N. Shackelford S. Harhay D. M. (2012). Biofilm formation by Shiga toxin-producing *Escherichia coli* O157:H7 and non-O157 strains and their tolerance to sanitizers commonly used in the food processing environment. J. Food Prot. 75, 1418–1428. doi: 10.4315/0362-028X.JFP-11-427 22856565

[ref63] Wang R. Kalchayanand N. King D. A. Luedtke B. E. Bosilevac J. M. Arthur T. M. (2014). Biofilm formation and sanitizer resistance of *Escherichia coli* O157: H7 strains isolated from “high event period” meat contamination. J. Food Prot. 77, 1982–1987. doi: 10.4315/0362-028X.JFP-14-253, PMID: 25364934

[ref64] Wang R. Luedtke B. E. Bosilevac J. M. Schmidt J. W. Kalchayanand N. Arthur T. M. (2016). *Escherichia coli* O157: H7 strains isolated from high-event period beef contamination have strong biofilm-forming ability and low sanitizer susceptibility, which are associated with high pO157 plasmid copy number. J. Food Prot. 79, 1875–1883. doi: 10.4315/0362-028X.JFP-16-113, PMID: 28221917

[ref65] Wickham G. (2017). An investigation into the relative resistances of common bacterial pathogens to quaternary ammonium cation disinfectants. Biosci Horizons 10:8. doi: 10.1093/biohorizons/hzx008

[ref66] Wood T. K. González Barrios A. F. Herzberg M. Lee J. (2006). Motility influences biofilm architecture in *Escherichia coli*. Appl. Microbiol. Biotechnol. 72, 361–367. doi: 10.1007/s00253-005-0263-8, PMID: 16397770

[ref67] Yang X. Wang H. He A. Tran F. (2018). Biofilm formation and susceptibility to biocides of recurring and transient *Escherichia coli* isolated from meat fabrication equipment. Food Control 90, 205–211. doi: 10.1016/j.foodcont.2018.02.050

[ref68] Yuan L. Wang H. Liu W. Sadiq F. A. Zhao Y. (2022). Editorial: multi-species biofilms in the food industry. Front. Microbiol. 13:428. doi: 10.3389/fmicb.2022.1023428, PMID: 36160223 PMC9504053

